# Socioeconomic Disparities in Iranians' Preferences toward Dental Care Services: A Population-Based Survey

**DOI:** 10.1155/2022/5652011

**Published:** 2022-10-26

**Authors:** Mohammad Khajedaluee, Zahra Yaghoubi, Tayebeh Malek Mohammadi, Kosar Sadat Hosseini

**Affiliations:** ^1^Department of Community Medicine, School of Medicine, Mashhad University of Medical Sciences, Mashhad, Iran; ^2^Department of Community Oral Health, School of Dentistry, Mashhad University of Medical Sciences, Mashhad, Iran; ^3^Social Determinants of Health Research Center, Institute for Futures Studies in Health, Department of Dental Public Health, Kerman, Iran; ^4^Student Research Committee, School of Dentistry, Mashhad University of Medical Sciences, Mashhad, Iran

## Abstract

**Background and Aims:**

Patients' perspectives and preferences are considered an essential influencing factor for healthcare utilization. This study is one of the first to investigate patient preference for dental services across socioeconomic and demographic indicators in Iran.

**Materials and Methods:**

This cross-sectional study was conducted through telephone interviews with adult residents in Mashhad and Kerman cities. A representative sample was selected by stratified random sampling. A valid structured questionnaire was used for data collection, including people's preference toward dental care services in terms of dental settings, choosing a general or specialist dentist, prevention or treatment, and the preferable gender of the dentist. Factors potentially associated with preferences included gender, age, educational level, job, monthly income, house size, family number, insurance coverage, dental insurance, type of insurance, and social class in the city were investigated.

**Results:**

1475 individuals participated in the study [response rate 63%]. Our findings showed higher preferences for private offices (50.6%), specialist dentists (76.2%), treatment services (40.8%), and no specific gender preference for the dentist (60.6%). Their preferences were significantly influenced by age range, social class, insurance status, dental insurance, and type of insurance. Income, household size, level of education, and job were not statistically significant with none of the preferences.

**Conclusions:**

Socioeconomic and demographic factors' correlation with people's preferences was observed. Efforts are needed to promote preventive care demand in deprived regions. Moreover, increasing financial resources allocated to preventive care and covering preventive dental services in insurance plans are recommended.

## 1. Introduction

Patients' perspectives and preferences are considered an essential influencing factor for health care utilization. Understanding people's views helps policymakers to more cost-effectively allocate available resources, meet patients' needs, and promote preventive health care utilization and cooperation with health care professionals [1–3]. Patients' preferences toward dental services are influenced by socioeconomic status, demographic background, cultural context, personality, and previous experiences [4, 5]. On the other hand, some evidence showed that health care providers' profiles, such as their ability and competence, gender, and nationality, besides the type of dental settings, services fee, insurance coverage, and insurance type are determinant factors too [6–8].

Understanding patients' perspectives on various aspects of dental services, such as where they receive dental care, the gender and specialty of the dentist, and the type of oral health services in terms of treatment or prevention, will help health policymakers make better decisions to plan health services [1, 2]. Some developed countries apply specific policies to maintain diversity in human resources according to the country's demographic characteristics [9]. Unfortunately, previous studies have mainly focused on patient's preferences in medical care, and little is known about patients' preferences for dental care [10].

Some studies have found no gender stereotype in getting dental care; controversially, other studies considered sex-concordance an influential factor [1, 6, 8]. This finding may be attributable to more comfortable patient-practitioner communication and less fear or shame during oral health procedures [11].

Currently, only 10% of Iranian dentists work in public health centers, and more than 80% of dental services are provided in the private sector [12]. This may make it difficult for those in the lower-income strata to access oral health services. A study in Canada showed that most Canadians prefer to seek dental care in a private setting. However, younger and disadvantaged patients are more likely to prefer public settings such as dental schools and community clinics [13]. As mentioned, insurance coverage and health insurance plans affect the patient oral health utilization. Some developed countries have uniform tariff policies in the private and public sectors, and their insurance plans cover various dental services, which significantly promote dental care utilization [14].

Understanding the health care consumers' views is necessary to promote the utilization of preventive oral health care. In Saadatfar's study in Iran, it was found that people mostly preferred restorative rather than preventive oral health procedures. Income and knowledge about preventive services associated with their preferences [15]. Preventive care is often ignored in low-income and developing countries, posing a tremendous economic burden [14]. There is no comprehensive evidence on patient preferences for dental care in Iran, and several questions have remained unanswered. Therefore, this study aimed to comprehensively assess people's preference toward dental care services in terms of types of services (prevention versus treatment), choosing a general or specialist dentist, type of dental setting, and preferable gender of the dentist across different socioeconomic and demographic backgrounds.

## 2. Materials and Methods

### 2.1. Study Design and Sampling

This cross-sectional telephone survey was conducted in Mashhad and Kerman, the two populous cities in the east of Iran. Adults aged 18 and over were eligible to participate in the study. Ethical approval was obtained from the Kerman University of Medical Science [number IR.kmu.REC.1394.549].

All the phone numbers were organized based on 35 inner urban city regions (28 regions in Mashhad, seven regions in Kerman) and 12 suburban areas (Mashhad 10, Kerman 2). Stratified random sampling was used to reach a representative sample. The calculated sample size (1475) was distributed based on each city's proportional population (Mashhad 1100, Kerman 375). Then, according to the proportion of telephone lines number in urban regions, the exact needed sample size was identified. Nonresponse and busy tell lines were excluded after two attempts. The Excel “RAND BETWEEN” function was used to gain a five-fold sample size to compensate for nonrespondent or busy phone lines.

### 2.2. Data Collection

A structured questionnaire was designed to evaluate participants' preferences. The validity and reliability of the questionnaire were assessed. Expect two omitted items; all questions obtained the acceptable content validity index (CVI) and content validity ratio (CVR). The reliability was assessed by the test-retest method, and Cronbach's alpha was acceptable too. The complete explanation process of the questionnaire development was reported in the previous study [16]. Three trained interviewers collaborated in this research. According to the pilot study, the interview process was revised, and the questionnaire was slightly modified. At the beginning of the interview, the participants were assured that the data would remain confidential and the identities of the individuals would not be revealed. The interviews were undertaken in the morning and evening of workdays from June to October 2016 by three. The detailed process of the main study is available in a previously published paper [17].

### 2.3. Background Variables (Demographic/Socioeconomic)

The demographic and socioeconomic profile of the population included gender, age, educational level (illiterate, elementary school, middle school, high school or diploma, associate degree, bachelor's degree, and over), job (employee, self-employed, worker, housewife, student, and unemployed); insurance coverage; having dental insurance; type of insurance (private and public), household size, monthly family income, and the social class in the city (affluent, middle, and disadvantage). Social class was determined based on the findings of a related study on the components of social status [18].

### 2.4. Outcome Variables

Participation's preferences were evaluated by the following questions:

1. Where do you usually get dental services? (1 = Private office, 2 = Private clinics, 3 = Charity clinics, 4 = Organization clinics, 5 = Public health centers, 6 = University educational clinics, 7 = combination of cases)
2. Do you prefer to go to a general dentist or a specialist? (1 = General, 2 = Special, No different)
3. Do you prefer to go to the dentist for preventive services or to go for treatment? (1 = Prevention, 2 = Treatment, 3 = Both)
4. Do you prefer female or male dentists? (1 = Female, 2 = Male, 3 = No different).

### 2.5. Data Analysis

Analytical and descriptive analyses were used to assess the sociodemographic characteristics of the respondents and their preferences by SPSS software (version 22). One-way analysis of variance (ANOVA) and the chi-square (*χ*^2^) tests were applied to evaluate the association between background and outcome variables.

## 3. Results

The overall response rate was 63% of 7291 phone calls made, 2854 were answered, 630 were commercial lines, 749 were not willing to participate, and finally, 1475 individuals participated in the study. 69.8% of respondents were females, and the subjects' mean age was 39 years (SD = 13.73); 35.3% held a university degree. About half of respondents were housewives (52.6%), and the majority were insured (88.8%); however, 48.1% did not have dental insurance. The detailed demographic data of participants are presented in Table 1.

According to the data analysis, income, household size, level of education, and job were not significantly associated with dental care preferences.

In terms of dental settings selection, half of the participants preferred private offices for receiving dental services (50.6%). Their attitude toward selecting each type of setting was significantly influenced by age range, social class, insurance status, having dental insurance (*p* < 0.0001), and type of insurance (*p* = 0.003). The private office was dominantly preferred among younger (under the age of 30) and elderly (over 60), upper social class persons, insured adults, people who had dental insurance and privately insured. The charity clinics were chosen as the second priority of the disadvantaged and those with no dental insurance.  Table 2 illustrates an overview of preferences for dental settings across different demographic and socioeconomic backgrounds.

As shown in Table 3, the majority of participants preferred specialist dentists (74.6%), which significantly correlated with age range (0.012), social class (*p* < 0.0001), being insured (0.033) and having dental insurance (*p* < 0.0001). Younger, higher social class, and insured participants, especially those with dental insurance, were more likely to prefer specialists. On the other hand, participants who had no specific preferences toward general or specialist dentists tended to be over 60 and disadvantaged.

Accordng to Table 4, Respondents were willing to receive treatment services over prevention (40% versus 22%). Participant's age range (*p* < 0.0001), social class (*p* < 0.0001), and dental insurance status (*p* = 0.003), had a significant impact on their preferences toward prevention or treatment services. Participants with dental insurance, youths, and affluent populations were more tendentious towards preventive services.

Over half of respondents reported that they had no specific gender preferences (60%). The participant characteristics associated with the dentist's gender selection were the type of insurance (*p* < 0.0001), social class in the city (*p* = 0.004), and respondent's gender (*p* < 0.0001). Participants with private insurance favored male dentists, while disadvantaged were more likely to prefer a female dentist. Patient preferences for the dentist's gender are compared in Table 5.

**Figure 1:**
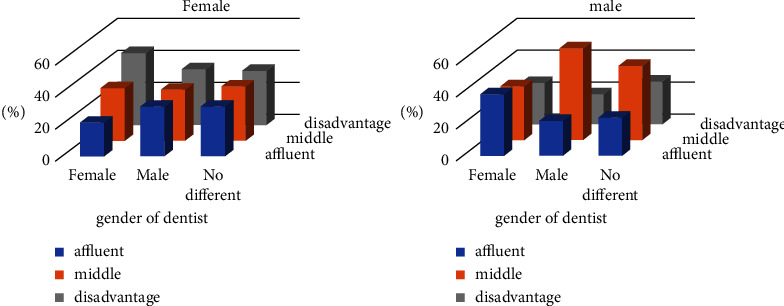
The difference in preferences by the participant's gender concerning their social class.

As presented in Figure 1, a notable interaction was found between social class and dentists' gender preference among female participants (*p* = 0.021). However, among males, this factor was not influential (*p* = 0.118). The female dentist was highly selected by social lower-class women, while the upper-class was less likely to prefer a female one.

## 4. Discussion

Our results cast a new light on Iranian adults' preferences toward dental services across different socioeconomic and demographic backgrounds.

The current study found that the majority of participants preferred the private sector over government setups, like in other studies in Saudi Arabia [10], Iran [4], and Canada [13]. People under the age of 30 and over 60 were more likely to prefer private dental institutions. According to other studies in Saudi Arabia [10] and Lithuania [19], older patients selected public sectors more often than younger ones; however, their finding is contrary to the study in Canada [13]. A possible explanation for this might be that younger age groups have more tendencies to elective aesthetic dental services such as orthodontic care and aesthetic restoration, which are dominantly delivered in the private sector. On the other hand, the elderly often require special care and attention, so providing a convenient appointment system is essential [20, 21]. There are similarities between the founded association of socioeconomic status and preferential healthcare settings and those described in studies in China [22], Saudi Arabia [20], and England [23], which partly imply health inequalities. These studies showed that the private sector is believed to provide better quality services, and as socioeconomic status improves, people are more willing to pay for luxury services. Also, they feel a more comfortable atmosphere in private offices. Being insured, dental insurance coverage, and private insurance coverage were essential determinants of dental setting preferences. In line with a previous study in Iran [4], privately insured persons and participants who had dental insurance favored the private sectors. In line with Saudi Arabia, dental insurance was not prevalent in the study [10]. Evidence showed that most insurance plans do not cover dental services in Iran, significantly influencing seeking needed dental services [24].

More than half of participants preferred specialist dentists, which was consistent with the study in Saudi Arabia [10]; however, according to a study in Australia and Sweden, 90% of both countries' respondents did not differentiate between a specialist and a general dentist who provides orthodontic treatment [25]. This contrast may be due to higher public trust toward practitioners in developed countries [26]. The younger and the higher social order participants have more tendencies to select a specialist. A study in Spain also confirmed the association between socioeconomic status and getting specialized services [27]. However, in Australia and Sweden, Youngers were more likely to prefer general dentists [25]. Since specialist dentists are known as skillful and more competent practitioners, it is necessary to educate patients about the role of specialists and improve patient satisfaction with care provided by the general dentist. Dental-insured individuals were more likely to prefer a specialist.

Our findings concerning different types of insurance were contrary to the study in Spain, which considered types of insurance as an influential factor on participants' choice [28]. This difference may be due to the different health insurance policies and coverage.

From the results, it is clear that most of the participants were in favor of treatment like in previous studies in Iran [15] and Bulgaria [29]. In contrast, in a study in Southern Thailand, parents' willingness to pay for sealants and fillings was not significantly different [30]. This dissimilarity is probably due to unawareness of the advantages of prevention and different health policies. In Iran, a higher proportion of decayed and missing teeth in the DMFT index may cause a higher preference toward curative services [31]. Youth and affluent participants were more likely to prefer prevention procedures. In the study of Tianviwat et al. in Thailand, younger parents compared to older ones had a higher tendency for preventive care for their children; however, the difference was not statistically significant [30]. According to studies in Greek and Brazil, residents with higher socioeconomic status had more frequent preventive dental checkups [32, 33]. The disadvantaged have fewer preventive dental visits. In addition to financial barriers, they are not sufficiently aware of the benefits of preventive care. Providing public oral health prevention care and increasing their awareness of its advantages is required for this group. Our finding indicated that having dental insurance increases the tendency for preventive care, like studies in Greek and the United States [32, 34], but the insurance type was not considered an influential factor, similar to Saadatfar's study in Iran [15].

More than half of the participants did not prefer the specific gender of the dentist, like studies in Saudi Arabia [7], the United States [35], and Sudan [36]. Among various factors, insurance type and social class was associated with their choice. Participants with private insurance were more likely to prefer male dentists. On the other hand, respondents of the lower social class tended to prefer female dentists, while in Alzahrani's study; participants considered cultural context, societal norms, and familial influences as the less effective factors on their preferences toward the dentist's gender comparison to other criteria [7]. Male and female participants' priority of the dentist's gender was statistically different across social classes. Compared to higher ones, the lower social class women preferred female dentists over male ones. A study in Canada showed that women with a preference for a female practitioner tended to be unemployed and have low social support [37]. It is not known why the upper social class females have fewer tendencies to choose the physician of the same gender. Future qualitative studies could further explore this issue. In our study, those under 30 and over 60 ages more frequently did not state gender stereotypes. In line with a study in Baghdad that found by increasing age, participants were more likely to have no specific gender preference; however, no significant association was found between respondents' age range and participant attitude in this regard [38].

Results showed that some socioeconomic variables, including income, household size, level of education, and job, were not statistically significant with none of the preferences. In contrast, in a study in China, income, age, and the number of family members were identified as significant factors [39]. Some other studies considered income an influential factor [4, 13, 30, 40]. Due to controversial results, more investigation is needed in this regard. It should be noted that people do not tend to spoil their income, and they may not state their income accurately.

The strengths of this study include the large sample size and assessing a wide range of socioeconomic and demographic characteristics. There were some limitations, too. Despite our efforts to achieve an equal gender distribution, the percentage of women was higher. On the other hand, self-reported socioeconomic status may not be valid and reliable [17]. So, we examined various socioeconomic indicators.

## 5. Conclusions

Our findings showed higher preferences for private offices, specialist dentists, treatment services, and low specific gender preference among the adult population of East Iran. Socioeconomic and demographic factors' correlation with people's preferences was observed. The findings propose the necessity of considering strategies to allocate resources in coordination with people's choices. Due to Iran's growing elderly population, health policymakers should consider their demands and desire to deliver appropriate dental services. Efforts are needed to promote preventive care demand in deprived regions. Moreover, increasing financial resources allocated to preventive care and covering preventive dental services in insurance plans are recommended.

## Figures and Tables

**Table 1:** Sociodemographic characteristics of the study population.

Study variables		Frequency (%)
*Gender*	Male	435 (30.2%)
	Female	1007 (69.8%)

*Age range*	<30	445 (30.3%)
	30–45	597 (40.6%)
	45–60	265 (18.0%)
	>60	164 (11.1%)

*Insurance*	Yes	1303 (88.8)
	No	165 (11.2%)

*Dental insurance*	Yes	384 (33.2%)
	No	557 (48.1%)
	Unknown	216 (18.7%)

*Education level*	Illiterate	57 (4.0%)
	Elementary school	176 (12.2%)
	Middle school	149 (10.4%)
	High school or diploma	549 (38.2%)
	Associates degree	112 (7.8%)
	Bachelor's degree and over	396 (27.5%)

*Job*	Employee	241 (17.0%)
	Worker	21 (1.5%)
	Self-employed	317 (22.4%)
	Student	67 (4.7%)
	Housewife	744 (52.6%)
	Unemployed	24 (1.7%)

**Table 2:** Participant's preferences for dental settings according to their demographic and socioeconomic characteristics.

Where do you prefer to get dental services?	Private office	Private clinics	Charity clinics	Organization clinics	Public health centers	University educational clinics	combination of all	Total	*p* Value (chi-square#)
Frequency (%)	698 (50.6%)	162 (11.7%)	187 (13.5%)	143 (10.3%)	65 (4.7%)	35 (2.5%)	107 (7.7%)	1379 (100%)	
*Age rang*									*p* < 0.0001
<30	50.6%	16.2%	13.3%	7.0%	5.8%	2.1%	4.8%	100% (413)	
30–45	50.4%	10.5%	14.4%	10.2%	4.2%	2.2%	7.7%	100% (567)	
45–60	42.9%	10.7%	14.0%	13.6%	4.5%	2.0%	11.9%	100% (242)	
>60	59.5%	3.5%	8.5%	13.4%	4.2%	4.9%	5.6%	100% (141)	

*Social class in the city*									*p* < 0.0001
Affluent	65.8%	7.7%	9.4%	8.9%	4.1%	4.1%	11.5%	100% (337)	
Middle	51.8%	13.0%	11.9%	11.5%	3.4%	2.0%	6.0%	100% (527)	
Disadvantage	41.4%	13.6%	18.9%	10.7%	7.2%	1.7%	6.1%	100% (454)	

*Insurance*									*p* < 0.0001
Yes	50.3%	10.5%	12.5%	11.1%	4.5%	2.7%	8.2%	100% (1238)	
No	46.45	20.9%	20.3%	2.6%	5.9%	0.7%	3.3%	100% (153)	

*Dental insurance*									*p* < 0.0001
Yes	48.9%	10.0%	7.8%	17.5%	6.7%	1.9%	7.2%	100% (630)	
No	51.7%	10.4%	14.7%	7.8%	3.0%	2.8%	9.7%	100% (538)	
Unknown	45.6%	12.1%	17.0%	8.7%	6.3%	3.4%	6.8%	100% (206)	

*Type of insurance*									0.003
Public	47.5%	10.9%	13.7%	11.5%	4.6%	3.1%	8.4%	100% (888)	
Private	61.8%	9.9%	5.4%	11.3%	3.9%	0.9%	6.4%	100% (202)	

**Table 3:** Participant's preferences for a specialist or general dentist according to their demographic and socioeconomic characteristics.

Do you prefer to go to a general dentist or a specialist for treatment?	General	Specialist	No different	Total	*p* Value (chi-square#)
Frequency (%)	50 (3.5%)	1101 (76.2%)	294 (20.3%)	1445 (100%)	
*Age rang*					0.012
<30	1.8%	80.8%	17.2%	100% (429)	
30–45	3.6%	76.2%	20.1%	100% (572)	
45–60	6.0%	72.8%	21.2%	100% (250)	
>60	3.1%	69.8%	27.3%	100% (157)	

*Social class in city*					*p* < 0.0001
Affluent	2.8%	83.1%	13.9%	100% (386)	
Middle	3.8%	76.1%	20.0%	100% (544)	
Disadvantage	3.7%	70.2%	25.9%	100% (474)	

*Insurance*					0.03
Yes	3.3%	77.2%	19.5%	100% (1275)	
No	4.9%	67.9%	27.2%	100% (162)	

*Dental insurance*					*p* < 0.0001
Yes	4.0%	80.2%	15.8%	100% (374)	
No	3.5%	78.8%	17.7%	100% (547)	
Unknown	1.9%	67.3%	30.8%	100% (214)	

*Type of insurance*					0.3
Public	3.5%	76.3%	20.1%	100% (910)	
Private	2.8%	81.3%	15.7%	100% (209)	

**Table 4:** Treatment/prevention services preference across demographic and socioeconomic respondents' characteristics.

Do you prefer to go to the dentist for preventive services or to go for treatment?	Prevention	Treatment	Both	Total	*p* Value (chi-square#)
Frequency (%)	320 (22.3%)	587 (40.8%)	530 (36.9%)	1437 (100%)	
*Age range*					*p* < 0.0001
<30	25.1%	34.4%	40.3%	100% (429)	
30–45	23.5%	38.6%	37.8%	100% (574)	
45–60	21.0%	45.3%	33.6%	100% (247)	
>60	10.0%	63.0%	26.8%	100% (149)	

*Social class in the city*					*p* < 0.0001
Affluent	27.6%	39.3%	33.0%	100% (384)	
Middle	23.5%	37.5%	38.9%	100% (544)	
Disadvantage	16.2%	47.1%	36.6%	100% (467)	

*Insurance*					0.44
Yes	22.6%	40.2%	37.2%	100% (1268)	
No	19.9%	45.3%	34.8%	100% (161)	

*Dental insurance*					0.003
Yes	26.6%	36.2%	37.0%	100% (373)	
No	20.9%	39.1%	40.0%	100% (545)	
Unknown	18.1%	50.5%	31.4%	100% (210)	

*Type of insurance*					0.08
Public	21.5%	41.5%	36.8%	100% (904)	
Private	27.9%	35.0%	36.9%	100% (211)	

**Table 5:** Patient preferences for dentist's gender.

Do you prefer female or male dentists?	Female	Male	No different	Total	*p* Value (chi-square#)		
					Female participant	Male participant	Total
Frequency (%)	270 (18.5%)	306 (20.9%)	886 (60.6%)	1462 (100%)			
*Age range*					0.95	0.21	0.98
<30	18.7%	19.6%	61.6%	100% (433)			
30–45	19.1%	21.0%	59.8%	100% (580)			
45–60	19.7%	21.7%	58.4%	100% (253)			
>60	17.0%	21.5%	61.3%	100% (158)			

*Social class in the city*					0.02	0.12	0.004
Affluent	15.6%	21.4%	62.9%	100% (391)			
Middle	16.3%	22.7%	62.6%	100% (549)			
Disadvantage	24.1%	17.9%	57.9%	100% (480)			

*Insurance*					0.16	0.43	0.27
Yes	18.1%	21.4%	60.5%	100% (1290)			
No	21.3%	16.5%	62.2%	100% (164)			

*Dental insurance*					0.88	0.65	0.77
Yes	18.7%	22.4%	58.8%	100% (379)			
No	18.7%	21.7%	59.6%	100% (552)			
Unknown	19.0%	18.1%	63.0%	100% (216)			

*Type of insurance*					0.004	0.04	*p* < 0.0001
Public	18.4%	19.1%	62.3%	100% (920)			
Private	16.0%	31.6%	52.3%	100% (212)			

## Data Availability

The data included in this paper are available from the corresponding author upon reasonable request.
